# Metastats: an improved statistical method for analysis of metagenomic data

**DOI:** 10.1186/gb-2011-12-s1-p17

**Published:** 2011-09-19

**Authors:** Joseph N Paulson, Mihai Pop, Hector Corrada Bravo

**Affiliations:** 1 Applied Mathematics and Scientific Computing Program, University of Maryland, College Park, MD 20742, USA; 2 Center for Bioinformatics and Computational Biology, University of Maryland, College Park, MD 20742, USA; 3 Department of Computer Science, University of Maryland, College Park, MD 20742, USA

## 

Metagenomic studies were originally focused on exploratory/validation projects but are rapidly being applied in a clinical setting. In this setting, researchers are interested in finding characteristics of the microbiome that correlate with the clinical status of the corresponding sample. Comparatively few computational/statistical tools have been developed that can assist in this process. Rather, most developments in the metagenomics community have focused on methods that compare samples as a whole. Specifically, the focus has been on developing robust methods for determining the level of similarity or difference between samples, rather than on identifying the specific characteristics that distinguish different samples from each other. Metastats [1] was the first statistical method developed specifically to address the questions asked in clinical studies. Metastats allows a comparison of metagenomic samples (represented as counts of individual features such as organisms, genes and functional groups) from two treatment populations (for example, healthy versus disease) and identifies those features that statistically distinguish the two populations.

Here, we present major improvements to the Metastats software and the underlying statistical methods. First, we describe new approaches for data normalization that allow a more accurate assessment of differential abundance by reducing the covariance between individual features implicitly introduced by the traditionally used ratio-based normalization. These normalization techniques are also of interest for time-series analyses or in the estimation of microbial networks. A second extension of Metastats is a mixed-model zero-inflated Gaussian distribution that allows Metastats to account for a common characteristic of metagenomic data: the presence of many features with zero counts owing to undersampling of the community. The number of ‘missing features’ (zero counts) correlates with the amount of sequencing performed, thereby biasing abundance measurements and the differential abundance statistics derived from them.

Using simulated and real data, we show that these methods significantly improve the accuracy of Metastats. We also describe the addition of several new statistical tests to our code (including presence/absence and the corresponding odds ratio, and penetrance calculations) that improve the usability of our software in clinical practice.
